# Hyperkinetic Movement Disorders in Children: A Brief Review

**Published:** 2019

**Authors:** Ali NIKKHAH, Parvaneh KARIMZADEH, Mohammad Mahdi TAGHDIRI, Mohammad Mahdi NASEHI, Mohsen JAVADZADEH, Elaheh KHARI

**Affiliations:** 1 Pediatric Neurology Research Center, Research Institute for Children’s Health, Shahid Beheshti University of Medical Sciences, Tehran, Iran; 2 2.Pediatric Neurology Department, Mofid Children’s Hospital, Faculty of Medicine, Shahid Beheshti University of Medical Sciences, Tehran, Iran

**Keywords:** Movement disorders, Hyperkinetic, Children, Pediatrics

## Abstract

Movement disorders are common neurologic disturbances in childhood. There are two major types of movement disorders. Hypokinetic disorders are with paucity of voluntary movements and are very uncommon in pediatric age group. Hyperkinetic movement abnormalities are very common in children and defined as abnormal repetitive involuntary movements. Movement disorders in childhood and even in adolescents are different in etiology, timing, treatment and prognosis versus adulthood movement abnormalities. In this brief article, we reviewed common types of hyperkinetic abnormal movements in children and adolescents with emphasis on etiologies, new classifications and recent treatment strategies.

## Introduction

Movement disorders are common neurologic disturbances in pediatric neurology and are one of the most common reasons for referral to pediatric neurology clinics (1). Childhood movement disorders are different in terms of etiology, timing, treatment, and prognosis toward adulthood movement abnormalities (1, 2). 

These disorders are characterized by impaired voluntary movements, the presence of involuntary movements, or both. They are dynamic disorders and their severity and distribution may shift over time (2, 3). Some involved children are unable to perform skilled motor plans and may be suffered from physical/social outcomes (3). 

Traditionally, movement disorders are classified to hyperkinetic and hypokinetic disorders. Hyperkinetic movement disorders are determined with abnormal repetitive involuntary movements (chorea, dystonia, athetosis, myoclonus, stereotypies, tics, and tremor). On the contrary, hypokinetic movement disorders revealed with reduced voluntary movements and akinesia (3, 4). Hypokinetic movement disorders are very unusual in pediatrics, therefore, in this review article, we dealt with some common hyperkinetic movement disorders in children with emphasis on etiologies, update classifications and new aspects of treatment. 

## Chorea

***Definition***

**Figure 1:**
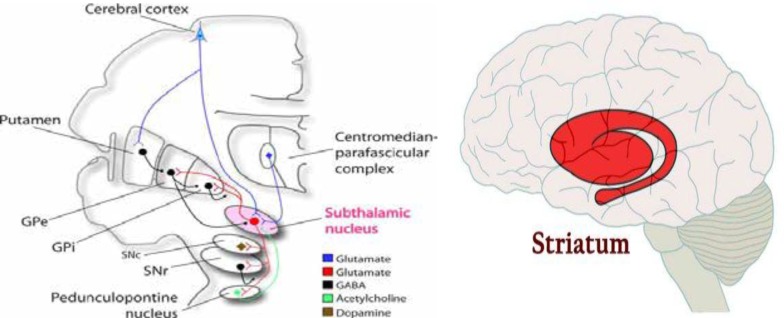
Striatum and subthalamic nucleus. Gpe: globus pallidus externus. Gpi: globus pallidus internus. SNe: substantia nigra compacta, SNr: Substantia nigra reticulata. (From canlabweb.colorado.edu)

Irregular, random, chaotic, brief and purposeless movements may flow from one part of body to another (5). These movements cannot be suppressed voluntarily (5, 6). Although most children with chorea have history of brain injuries but rare genetic causes should be considered (6). Athetosis is slower and writhing form of chorea with involvement of distal part of extremities more than proximal. Conversely, Ballismus is high-amplitude, forceful and flinging form of chorea that mainly involves proximal joints and muscles (5-7). Pathophysiologically, chorea typically occurs due to dysfunction of the striatum or subthalamic nucleus (1, 5) (Figure 1). 

## Etiology

There are numerous causes of chorea. Some of more common etiologies are (6-8)

- **Static/Structural:** Cerebral palsy, stroke, cerebral vasculitis, brain tumors.
- **Hereditary/Degenerative**: Pantothenate kinase-associated neurodegeneration (PKAN), Fahr disease, Ataxia-telangiectasia.
- **Metabolic: **Niemann-Pick type C, propionic acidemia, methylmalonic aciduria, mitochondrial disorders.
- **Infectious/Parainfectious:** Encephalitis, postencephalitis.
- **Immune-Mediated/Demyelinating:** Sydenham Chorea, SLE.
- **Drugs/Toxins: **Neuroleptics, antiseizure drugs, stimulants, clonidine.
- **Paroxysmal Disorders:** Migraine, paroxysmal kinesigenic dyskinesia.

## Dystonia

***Definition***

**Figure 2:**
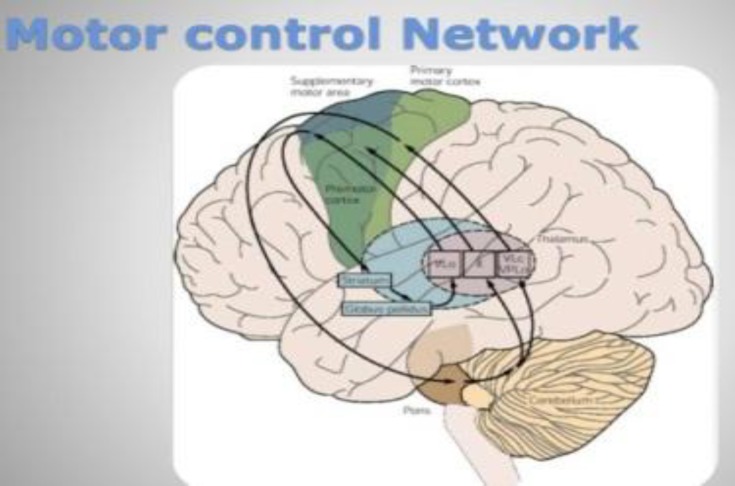
Motor control network (from natural neural network.org)

Involuntary muscular contractions cause painful or uncomfortable twisting and repetitive movements, abnormal intermittent fixed postures or both (9). In fact, dystonia is due to simultaneous agonist-antagonist muscles contraction (10). This abnormal movement may exacerbate by voluntary actions, stress, fatigue, and pain. Dystonia often decreases or disappears at sleep (10, 11). Historically dystonia arises from basal ganglia (especially globus pallidus) but, nowadays, any injury or dysfunction in motor control network (basal ganglia, cortex, internal capsule, thalamus, brainstem, and cerebellum) can produce dystonia and other movement disorders (12) (Figure 2). 

Etiology 

Some more common etiologies of dystonia in children are (9, 10)

**Static/Structural:** Cerebral palsy, kernicterus, hypoxic-ischemic injury, stroke.

- **Hereditary/Degenerative**: DYTx (x=1 to 16) Dystonia, Leigh syndrome, PKAN, Niemann-Pick type C, striatal necrosis.
- **Metabolic:** Glutaric aciduria, mitochondrial disorders, Wilson disease.
- **Drugs/Toxins: **Same as chorea.
- **Paroxysmal Disorders:** Same as chorea.

New Classification (1, 11, 13)

**Axis I. Clinical characteristics:**

- **Age at onset:** Infancy (birth to 2 yr), childhood (3 to 12 yr), adolescence (13 to 20 yr), early adulthood (21 to 40 yr), late adulthood (> 40 yr).
- **Body distribution: **Focal (Segmental, Multifocal), generalized.
- **Temporal pattern: **Disease course (Static, Progressive), variability (Persistent, Action-specific, Diurnal, Paroxysmal).

**Axis II. Etiology:**

- **Nervous system pathology: **Evidence of degeneration, Evidence of structural lesions, No evidence of degeneration or structural lesion.
- **Inherited or acquired: **Inherited (autosomal dominant, autosomal recessive, x-linked recessive, mitochondrial), Acquired (perinatal brain injury, infections, drugs, toxins, neoplasm, vascular).
- **Idiopathic: **Sporadic, familial.

## Stereotypies

Abnormal, repetitive, purposeless, rhythmic, patterned, episodic movements demonstrate into constant form in over time (14, 15). Other names of this abnormal movement are "self-stimulation", "gratification phenomena" and "rhythmic habit pattern" (14-16). These movements almost always aren’t seen in lower extremities and usually occurred in head and distal part of upper extremities. Stereotypies can occur in neurologically impaired patients or in otherwise normal children. Common underlying etiologies associated with stereotypies are autistic spectrum disorders, intellectual disabilities and sensory deficits (15-17). Some more common these abnormal movements include head banging, body rocking, thumb sucking, facial grimacing, waving and wrist rotation (14-16). Fortunately, stereotypies do not interfere with routine daily activities of involved children and could be ceased by distraction or engaging in a new activity. Stereotypies can be exacerbating by fatigue, stress and excitement (17-19).

**Tics**

***Definition***

**Figure 3:**
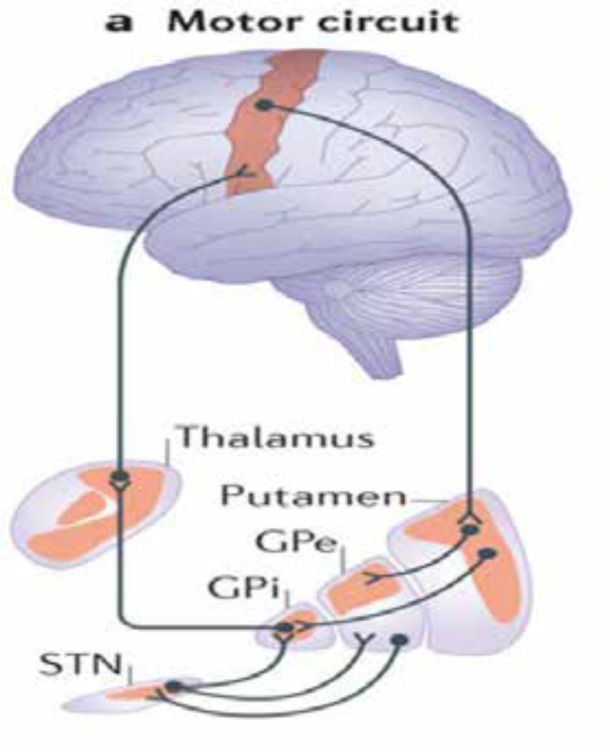
Cortico-striato-thalamic-cortex loop. STN: Subthalamic nucleus (from Nature Reviews Neuroscience).

Tics as the most common movement disorder in childhood, are involuntary, rapid, abrupt, repetitive, recurrent, and nonrhythmic movements or vocalizations. Tics are suppressible and almost always disappear at sleep and can be exacerbating with stress, excitement and anxiety (20-22). This type of abnormal movements is subdivided into simple and complex categories. Traditionally, tics were classified to motor and vocal (phonic) groups, whereas vocal tics are the result of muscle contraction of diaphragm and oropharynx, this formal classification has been questionable recently. In fact, all tics have muscular origin (21, 22). Tourette syndrome or disease is the most severe and disabling chronic form of tic disorder that usually comorbid with attention deficit hyperactivity disorder (ADHD) and obsessive-compulsive disorder (OCD) and can interfere with normal daily activities (23, 24). Pathophysiologically, any abnormality of neurotransmitters or structures in cortico-striato-thalamic-cortex loop (motor circuit) can be leading to tic disorders (21) (Figure 3).

Tic Phenomenology

**Figure 4:**
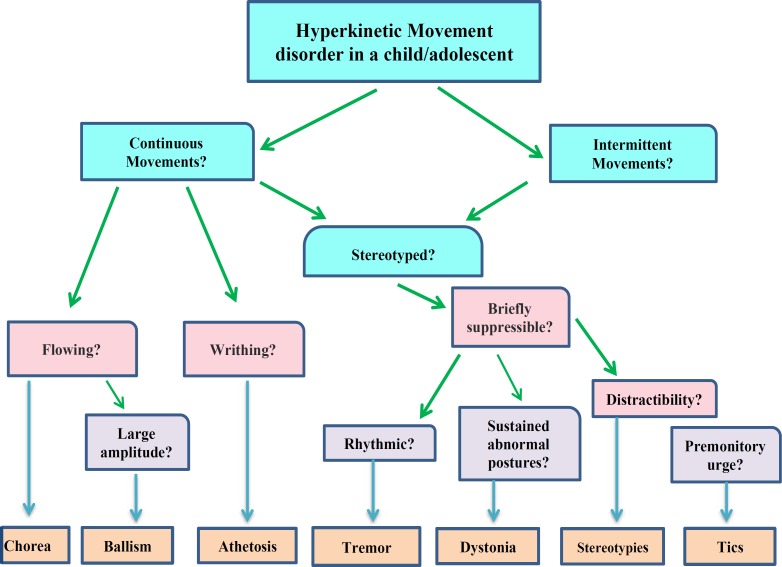
A simple pragmatic algorithm for approach to hyperkinetic movement disorders in children (Modified form of algorithm from cpamm.asc.org)

**Simple motor tics: **Involve single muscle group (eye blinking, nose wrinkling, shoulder shrugging, and abdominal tensing) (20, 21, 23).

- **Complex motor tics: **Are more purposeful and complicated than simple tics (echopraxia, and copropraxia).** Echopraxia: **Repeating other person's movements. **Copropraxia:** repeating socially inappropriate gestures.
- **Simple vocal tics: **Simple sounds and noises (throat clearing, sniffing, yelps, screech, and grunts).
- **Complex vocal tics: **Repetition of words or phrases with special vocalization (palilalia, echolalia, coprolalia). **Palilalia:** repeating one's own words. **Echolalia:** repeating other person's word. **Coprolalia:** repeating obscene words.

New Classification (DSM V) (21, 23)

- **Provisional tic disorder: **Is known with simple motor and/or vocal tics that begin before age 18 yr and have been present less than 12 months.
- **Chronic tic disorder: **As provisional tics except be continued more than 12 months.
- **Tourette syndrome: **Is distinguished by multiple motor tics that may be occurring with at least one vocal tic simultaneously in a specified period of time (usually 1 year).
- **Substance-induced tic disorder: **Tics occur at least during or within one-month substance intoxication or withdrawal. The most common substances associated with tics are cocaine and neuroleptics.
- **Tic disorder due to generalized medical condition: **Some medical conditions such as Huntington disease, neurodegeneration with brain iron accumulation, stroke, encephalitis, and even head trauma.
- **Tic disorder not otherwise specified: **Refers to tic disorders not classified in other groups. These tics are unusual in onset and presentation.

## Tremor

***Definition***

Tremor is defined as oscillatory, involuntary, regular, and rhythmic movements of body parts. Tremor can affect head, extremities, trunk and even soft palate separately or combined (25, 26). Frequency of oscillations around a joint can vary with change of position. Tremors are relatively common in adolescents and fine motor activity of involved persons can be limited by severe sustained tremors (26, 27). Pathophysiologically, any dysfunction of cortex, basal ganglia, brainstem and cerebellum can cause tremor (26). 

**Etiology**

The most common causes of tremor in children and adolescents are as follows (26, 27)

**Benign tremors:** Enhanced physiologic tremor, and shuddering attacks.

- **Static injury/Structural disorders: **Stroke (especially in midbrain), and multiple sclerosis.
- **Hereditary/Degenerative disorders: **Familial essential tremor, juvenile Parkinson, and Wilson disease.
- **Metabolic disorders: **Hyperthyroidism, hypoglycemia, hypocalcemia, and hepatic encephalopathy.
- **Drugs/Toxins: **Valproate, lithium, stimulants, neuroleptics, arsenic, mercury, lead, and ethanol.
- **Other causes:** Cerebellar disorders, and functional tremor.

Types of tremor 

**Rest tremor: **The frequency of this type is between 4 and 6 Hz. This tremor can be attenuated or resolved with movements or antigravity position and usually worsens with stress and agitation. Tremor at rest origins from any dysfunction of basal ganglia (especially substantia nigra) (25-29).

- **Action tremor: **This type of tremor can be started or exacerbated by movements. There are four subtypes of action tremor:

1. **Kinetic tremor: **Can occur with any voluntary movement and are uniform.
2. **Intention tremor: **Worsens with the end of targeted movements and originates from cerebellar dysfunctions.
3. **Isometric tremor: **Can produce by additive force against fixed target.
4. **Postural tremor:** Occurs with maintaining a body part in a constant immobile position. This type is due to Cerebello-Olivary system dysfunction.

- **Rubral tremor: **Named as Holmes or midbrain tremor. This type of tremor is distinguished by coarse, large amplitude and low-frequency irregular jerky movements. It is usually nonprogressive but may be very disabling. Any structural or functional abnormality in midbrain and thalamus can cause this type of tremor.
- **Physiologic tremor:** There are normal motor oscillations in humans. This normal condition can be enhanced or exacerbated with some situational challenges such as; excitement, fatigue, and caffeine consumption.
- **Psychogenic tremor: **Is an acute onset, nonprogressive tremor that is due to underling psychiatric condition.

## Treatment strategies

***General aspects***

Nowadays, treatment of movement disorders should be performed based on the symptomatology independent of underlying condition (2, 4). In fact, we want to disrupt the connection between pathophysiological cause and clinical presentation on basis of neurotransmitters' functions. On the other hand, we have to try to reduce personal/social disabilities due to abnormal movements with pharmacologic with or without supportive therapy (2, 4, 13). Moreover, we should recognize the main etiology of abnormal movement and other underlying conditions for decision of the best treatment strategy. 

***Specific treatment***

- **Chorea/Athetosis: **Some drugs have effect on chorea and can reduce its severity such as valproate, carbamazepine, benzodiazepines, tetrabenazine, reserpine and neuroleptics (2, 4, 30). Neuroleptics are potent anti-chorea drugs and are suitable for severe chorea with irreversible etiologies and patients suffering behavioral problems (4, 30). Some kinds of chorea especially paroxysmal kinesigenic chorea response to carbamazepine, dramatically (31). Recently, some new selective vesicular monoamine transporter (VMAT) blockers (methoxytetrabenazine, dihydrotetrabenazine, and valbenazine) are developed and have very low side effects (32, 33). Deep brain stimulation (DBS) can be used in refractory cases (30, 33).
- **Dystonia: **Some common forms of primary dystonia are dopa-responsive and starting a trial of L-DOPA for all children with unexplained dystonia is reasonable (4, 13, 34). Anticholinergic drugs are effective in many types of dystonia (2, 13, 34). Deep brain stimulation may be helpful in some primary and secondary forms of dystonia (2, 4, 34,35). Other drugs contain botulinum toxin (for some focal dystonia), and benzodiazepines (13, 35). Oral baclofen (for painful dystonia), intrathecal baclofen (for combined dystonia and spasticity) (13, 36).
- **Tics: **Despite advances in understanding of tic mechanisms, there is not any drug can suppress tics completely yet (21, 22, 37). In fact, our goal of treatment is reduction of severe frequent tics and helping to better quality of life subsequently (38, 39). Behavioral cognitive therapy is mandatory for management of tics but some severe cases or patients with comorbidities need to pharmacologic therapy (39, 40). Tier 1 medications (clonidine, guanfacine) have been used for mild tics especially with comorbid ADHD. Tier 2 medications contain typical (halopridole, pimozide, fluphenazine) and atypical (risperidone, aripiprazole, ziprasidone, quetiapine) neuroleptics can be prescribed for severe tics (37-40). Tetrabenazine and clonazepam have minor effect on some refractory tics (32, 37). Finally, DBS can be considered in refractory tics especially in Tourette syndrome though there is not enough evidence for effectiveness of DBS in children (37, 41).
- **Tremor:** Treatment of tremor needs to definite exclusion of causing-drug tremor, cerebellar dysfunctions, metabolic, and hormonal causes (25). There are some drugs for essential and exaggerated physiologic tremors such as; propranolol, primidone, clonazepam, gabapentin, and topiramate (25, 26, 27). Botulinum toxin injections can be used for refractory head and voice tremors (27, 28). Holms tremor is refractory to these drugs although, some involved persons respond to clonazepam, L-DOPA (42). Ultimately, thalamic DBS can be used for some drug-resistant disabling tremors (27, 28). Recently, octanoic acid has been used for severe essential voice tremors (43).

**In conclusion, **hyperkinetic movement disorders are very common in children and review of definitions, new classifications and update treatment strategies is mandatory for pediatric neurologists and psychiatrists. Ultimately, we drew a brief practical algorithm as pragmatic abstract of this article for initial appropriate clinical approach (Figure 4). 

## References

[1] Saunders-Pullman R, Braun I, Bressman S (1999). Pediatric movement disorders. Child Adolesc Psychiatr Clin North Am.

[2] Singer HS, Mink JW, Gilbert DL ( 2016). Movement Disorders in Childhood, second ed.

[3] Sanger TD, Chen D, Fehlings SL, Hallett M, Lang AE, Mink JW (2010). Definition and classification of hyperkinetic movements in childhood. Mov Disord.

[4] Dale RC, Singh H, Troedson C, Pillai S, Gaikiwari S, Kozlowska K (2010). A prospective study of acute movement disorders in children. Dev Med Child Neurol.

[5] Cardoso F, Seppi K, Mair KJ, Wenning GK, Poewe W (2006). Seminar on choreas. Lancet Neurol.

[6] Pandey S (2013). Chorea. J Assoc Physicians India.

[7] Wild EJ, Tabrizi SJ (2007). The differential diagnosis of chorea. Pract Neurol.

[8] Gilbert DL (2009). Acute and chronic chorea in childhood. Semin Pediatr Neurol.

[9] Albanese A, Bhatia K, Bressman SB, Delong MR, Fahn S, Fung VS (2013). Phenomenology and classification of dystonia: A consensus update. Mov Disord.

[10] Geyer HL, Bressman SB (2006). The diagnosis of dystonia. Lancet Neurol.

[11] Batla A (2018). Dystonia: A review. Neurol India.

[12] Neychef VK, Gross RE, Lehericy S (2011). The functional neuroanatomy of dystonia. Neurobiol Dis.

[13] Macerollo A, Martino D (2016). What is new in tics, dystonia and chorea?. Clin Med (Lond).

[14] Mahone EM, Bridges D, Prahme C, Singer HS (2004). Repetitive arm and hand movements (complex motor stereotypies) in children. J Pediatr.

[15] Muthugovindan D, Singer H (2009). Motor stereotypy disorders. Curr Opin Neurol.

[16] Kennedy CH, Meyer KA, Knowles T, Shukla S (2000). Analyzing the multiple functions of stereotypical behavior for students with autism: Implications for assessment and treatment. J Appl Behav Anal.

[17] Goldman S, Wang C, Salgado MW, Greene PE, Kim M, Rapin (2009). Motor stereotypies in children with autism and other developmental disorders. Dev Med Child Neurol.

[18] Freeman RD, Soltanifar A, Baer S (2010). Stereotypic movement disorder: easily missed. Dev Med Child Neurol.

[19] Singer HS (2011). Stereotypic movement disorders. Handb Clin Neurol.

[20] Cohen, SC, Leckman, JF, Bloch, MH (2013). Clinical assessment of Tourette syndrome and tic disorders. Neurosci Biobehav Rev.

[21] Plessen KJ (2013). Tic disorders and Tourette's syndrome. Eur Child Adolesc Psychiatry.

[22] Knight T, Steeves T, Day L, Lowerison M, Jette N, Pringsheim T (2012). Prevalence of Tic Disorders: A Systematic Review and Meta-Analysis. Pediatr Neurol.

[23] Blackburn JS (2018). Tic Disorders and PANDAS. Semin Pediatr Neurol.

[24] Doja A, Bookwala A, Pohl D, Rossi-Ricci A, Barrowman N, Chan J, Longmuir PE (2018). Relationship between Physical Activity, Tic Severity and Quality of Life in Children with Tourette syndrome. J Can Acad Child Adolesc Psychiatry.

[25] Louis ED, Dure LS, Pullman S (2001). Essential tremor in childhood: A series of nineteen cases. Mov Disord.

[26] Keller S, Dure LS (2002). Tremor in childhood. Semin Pediatr Neurol.

[27] Prasad M, Ong MT, Whitehouse WP (2014). Fifteen minute consultation: tremor in children. Arch Dis Child Educ Pract Ed.

[28] Raina GB, Cersosimo MG, Folgar SS, Giugni JC, Calandra C, Paviolo JP (2016). Holmes tremor: Clinical description, lesion localization, and treatment in a series of 29 cases. Neurology.

[29] Lenka A, Louis ED (2018). Revisiting the Clinical Phenomenology of "Cerebellar Tremor": Beyond the Intention Tremor. Cerebellum.

[30] Feinstein E, Walker (2018). An Update on the Treatment of Chorea. Curr Treat Options Neurol.

[31] Bhatia KP (2011). Paroxysmal dyskinesias. Mov Disord.

[32] Paleacu D, Giladi N, Moore O, Stern A, Honigman S, Badarny S (2004). Tetrabenazine treatment in movement disorders. Clin Neuropharmacol.

[33] Bashir H, Jankovic J (2018). Treatment options for chorea. Expert Rev Neurother.

[34] Luc QN, Querubin J (2017). Clinical Management of Dystonia in Childhood. Paediatr Drugs.

[35] Albanese A, Di Giovanni M, Lalli S (2019). Dystonia: diagnosis and management. Eur J Neurol.

[36] Albright AL, Barry MJ, Shafton DH, Ferson SS (2001). Intrathecal baclofen for generalized dystonia. DEV Med Child Neurol.

[37] Qasaymeh MM, Mink JW (2006). New treatments for tic disorders. Curr Treat Options Neurol.

[38] Wu SW, Harris E, Gilbert DL (2010). Tic suppression: the medical model. J Child Adolesc Psychopharmacol.

[39] Ganos C, Martino D, Pringsheim T (2017). Tics in the Pediatric Population: Pragmatic Management. Mov Disord Clin Pract.

[40] Pringsheim T (2017). Tic Severity and Treatment in Children: The Effect of Comorbid Attention Deficit Hyperactivity Disorder and Obsessive Compulsive Behaviors. Child Psychiatry Hum Dev.

[41] McGuire JF, Piacentini J, Brennan EA (2014). A meta-analysis of behavior therapy for Tourette syndrome. J Psychiatr Res.

[42] Aydın S, Canaz H, Tuna Erdogan E, Durmaz N, Topcular B (2017). Holmes’ Tremor with Shoulder Pain Treated by Deep Brain Stimulation of Unilateral Ventral Intermediate Thalamic Nucleus and Globus Pallidus Internus. J Mov Disord.

[43] Lowell SY, Kelley RT, Monahan M, Hosbach-Cannon CJ, Colton RH, Mihaila D (2018). The Effect of Octanoic Acid on Essential Voice Tremor: A Double-Blind, Placebo-Controlled Study. Laryngoscope.

